# Discovery of ROCK2 inhibitors through computational screening of ZINC database: Integrating pharmacophore modeling, molecular docking, and MD simulations

**DOI:** 10.1371/journal.pone.0323781

**Published:** 2025-05-13

**Authors:** Abdullah R. Alanzi, Abdelaaty A. Shahat, Bayan Abdullah Alhaidhal, Raghad Mohammad Aloatibi

**Affiliations:** Department of Pharmacognosy, College of Pharmacy, King Saud University, Riyadh, Saudi Arabia; Bowen University, NIGERIA

## Abstract

Rho-associated protein kinase 2 (ROCK2) is a serine/threonine kinase that is crucial for regulating various physiological processes and is part of the Rho-associated coiled-coil kinase family. The dysregulation of ROCK2 has been associated with a range of diseases, making it a promising target for therapy. In this study, a chemical feature-based pharmacophore model was developed on the co-crystal ligand (5YS) of ROCK2 to conduct the virtual screening of ZINC database, resulting in 4809 hits that were further subjected to molecular docking to find the binding affinities with ROCK2 protein. The binding affinities of the hits were analyzed and compounds in the range of -11.55 to -9.91 kcal/mol were selected for further analysis. The ADMET analysis identified two promising compounds, whose binding stability with the ROCK2 protein was further evaluated using molecular dynamics (MD) simulations. Simulation results revealed that the selected compounds remained closely bound to protein indicating that they can act as lead compounds to control the biological activity of ROCK2. However, further in vitro investigation is required to test the biological efficacy of the reported compounds.

## 1. Introduction

Serine/threonine kinase Rho-associated protein kinase 2 (ROCK2) regulates the actin cytoskeleton and affects cell shape, adhesion, migration, and proliferation. It is involved in many biological processes [1]. ROCK2 activation is tightly regulated by the small GTPase RhoA, which binds to ROCK2 and relieves auto-inhibition, resulting in increased kinase activity [2]. ROCK2 is an intracellular expression of Rho-associated coiled-coil containing kinases, one of the two mammalian homologs. It is found in muscle cells, neurons, and the kidney and bladder epithelium [2,3]. ROCK2 has been linked to a variety of cellular functions such as actin organization, neuronal growth cone guidance, cell migration, synaptic transmission, and cancer cell invasion and proliferation. The metastatic phase of several malignancies, including melanoma, bladder, liver, and breast cancer, is linked to the ROCK2 pathway [4,5]. ROCK2 expression has been linked to tumor invasion, metastasis, and a poor prognosis in bladder and renal cancers [6].

It has recently been determined that the ROCK pathway plays a role in both neuronal degeneration and regeneration [7]. Increased intrinsic ROCK-activity in neurons severely disrupts the growth cone machinery, impeding regenerative processes. The anti-inflammatory M2-state is maintained over the pro-inflammatory M1-phenotype by ROCK signaling [1]. The RhoA/Rho-kinase pathway has been shown to be important for a number of vital cellular functions, with ROCK2 being mainly responsible for vascular smooth muscle cell contraction [8]. Several reported studies suggests that ROCK2 may serve as a therapeutic target for treating neurodegenerative disorders such as Huntington’s disease (HD), Parkinson’s disease (PD), and Alzheimer’s disease [9–13]. The overexpression ROCK2 decreases the rate of Parkin recruitment, which is important for the neuro-protective Parkin-mediated mitophagy pathway. So, the over-expression of ROCK2 contributes to the development of PD by decreasing the rate of Parkin recruitment and increasing α-synuclein pathology [14]. ROCK inhibitors, such as Fasudil, have been shown to attenuate these effects and have potential as disease-modifying drugs for the treatment of PD [15]. The discovery of ROCK2 inhibitors generally holds promise for both postponing the onset of neurodegeneration and promoting neurodegeneration in a variety of neurological conditions.

ROCK inhibitors (ROCKIs) have been identified so far in several classes with varying scaffolds, such as is quinolines, pyridines, amides, benzo dioxane pyrazoles, indazoles, and urease [16–18]. Only Fasudil, one of many ROCK inhibitors, has been available on the Japanese market since 1995 for the management of cerebral vasospasm and ischemia. Compound H-1152P is derived from Fasudi and is a dimethyl-fasudil [19,20]. Similar to fasudil, Y-27632 has been shown to bind to bovine ROCK2 co-crystal structure and exhibit superior potency and selectivity against the enzyme [21]. The compounds with pyrazole heterocyclic rings (SR6074 and SR6494) and benzothiazole2-carboxamide substitutions were thought to be the most effective ROCK2 inhibitors [22].

Our study presents a state‐of‐the‐art integrative computational pipeline that combines pharmacophore modeling, virtual screening, molecular docking, ADMET profiling, and molecular dynamics simulations to identify potent ROCK2 inhibitors. By systematically mining the extensive ZINC database and rigorously filtering candidates based on binding affinity and drug-like properties, our methodology not only ensures that the selected compounds demonstrate strong and stable interactions with key active site residues of ROCK2 but also optimizes their pharmacokinetic profiles for potential therapeutic application. The incorporation of molecular dynamics simulations further confirms the structural stability and dynamic behavior of the ligand–protein complexes, highlighting the reliability of our predictions. This multi-tiered approach is superior to conventional screening methods as it reduces experimental time and costs while significantly enhancing the accuracy of lead identification. Ultimately, our innovative framework offers a promising solution for the development of targeted ROCK2 inhibitors, which could play a crucial role in mitigating the progression of diseases associated with ROCK2 dysregulation, such as cancer metastasis, cardiovascular disorders, and neurodegenerative conditions.

The search for ROCK2 inhibitors in wet-lab research is a labor-intensive and time-consuming procedure. Computational approaches significantly enhance the early-stage identification and screening of potential ROCK2 inhibitors, reducing time and cost in drug discovery. The benefits of these computational techniques have been addressed in several published successful applications to date [21,23,24]. This study focuses on the computational screening of the ZINC database to find putative ROCK2 inhibitors by utilizing computational drug design approaches.

## 2. Methodology

### 2.1 Pharmacophore modelling

A pharmacophore model can be described as a template comprising of the essential chemical features of biologically active compounds. The pharmacophoric features of active compounds are utilized to generate pharmacophore model which then processed to conduct the screening_Fg of large chemical databases [25]. We developed a pharmacophore model using the chemical features of a co-crystal ligand (5YS) of ROCK2 protein (PDB ID: 7P6N) by utilizing the Pharmit server [26,27]. The pharmacophore model was created by analyzing its interactions within the ROCK2 binding pocket.

### 2.2 Virtual screening

Four pharmacophoric features aromatic ring, hydrophobic group, hydrogen bond donor and acceptor of the co-crystal ligand 5YS were used to generate the pharmacophore hypothesis. The parameters to screen the compounds from databases adhered to Lipinski’s rule [28], specifying that molecular weight should be less than 500, HBD less than 5, HBA less than 10, and logP less than 5. For the virtual screening, ZINC databases containing 13,127,550 molecules were explored.

### 2.3 Ligand preparation

A total of 4809 hits obtained from virtual screening were processed using the LigPrep program in Schrödinger’s Maestro [29]. OPLS_2005 force field was used to optimize the geometry of the ligands, ensuring they achieved energetically favorable conformations [30]. Energy minimization was applied to remove any unfavorable interactions or strained geometries.

### 2.4 Molecular docking

The prepared screened compounds were used in the docking of ROCK2 receptor. The x-ray crystallographic structure of ROCK2 was retrieved from PDB database (PDB ID: 7P6N) and prepared for the docking using Protein Preparation Wizard [31]. Before proceeding towards the receptor preparation, the missing loops of the protein were modeled using the Modeller tool [32]. During receptor preparation, several stages were done including the generation of disulfide bonds, assignment of zero-order metal bonds, and addition of hydrogens. The additional ligands and crystal water were also removed. In the optimization step, the pKa values of ionizable group were optimized at pH 7.0 utilizing the PROPKA program [33]. Finally, the OPLS_2005 force-field was used for energy minimization. After the protein preparation, a three-dimensional grid was constructed at Cartesian coordinates with values of 83.45, 40.13, and 18.05 for X, Y, and Z respectively, for site specific docking. The screened compounds were docked to the protein using SP mode of glide [34].

### 2.5 Toxicity analysis

The drug erosion is linked to toxicity concerns and suboptimal pharmacokinetics of the compounds [35]. To address this, the ADMET profiles were analyzed to evaluate the toxicity risks of drug candidates [36]. This predictive approach also helps in evaluating the likelihood of lead compounds becoming viable oral drugs. In this study, we used OSIRIS Property Explorer tool [37] to predict the ADMET characteristics of the most promising compounds. We assessed several pharmacokinetic properties, including molecular weight (MW), solubility (log S), logP, TPSA, and drug-likeness and score. Additionally, we scrutinized the compounds for potential toxicity consequences, encompassing tumorigenic, mutagenic, irritating, and reproductive concerns.

### 2.6 MD simulation

To analyze the protein ligand stability, a MD simulation for 150 ns was executed on the complexes of C2 compound and C4 compound. The solvation of the complex was done in a periodic box with a 10 Å size containing the TIP3P water molecules [38]. Counter ions of Na + and Cl- were introduced into the system to neutralize it. The minimization of the system was performed using the steepest decent method of 5000 steps following neutralization to remove steric conflicts. After minimization, the systems were prepared for the production run by equilibrating for 50,000 and 100,000 steps, respectively, at 310 K temperature at the NVT and NPT ensembles [39]. The simulation was Conducted with the Berendson thermostat and Parrinello-Rahman algorithms to maintain a constant temperature (310 K) and pressure (1 ATM). By adjusting the time at τ P = 2.0 ps and τ T = 0.1 ps, the system was relaxed, and by applying the LINCS algorithm, the hydrogen atoms’ bond lengths were kept at their ideal lengths [40], whereas Verlet computed the non-bonded interactions [41]. To compute the electrostatic interactions beyond the short-range limit, the particle mesh Ewald approach was used [42]. In x, y, and z dimensions, the Periodic boundary conditions were imposed, and a production run was conducted on the system. Every 10ps, the production run’s trajectory was saved and examined using the R BIO3D package and gromacs commands [43] CHARMM36 force-field and the Gromacs simulation program were used to execute the simulation [44].

## 3. Results

### 3.1 Virtual screening

**Fig 1:**
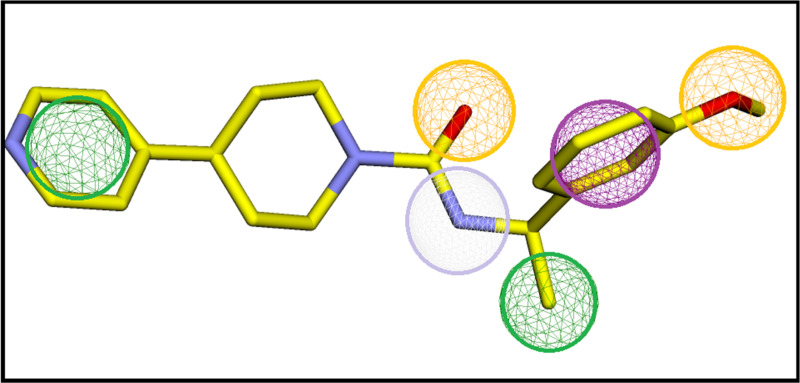
The pharmacophore hypothesis. Green spheres represent hydrophobic groups, purple indicates aromatic rings, gray denotes hydrogen bond donors, and orange spheres signify hydrogen bond acceptors.

The pharmacophore hypothesis was developed on the 5YS ligand atoms that interacted with the ROCK2 protein. The hypothesis comprised six features (Fig 1). The cartesian coordinates of these features are detailed in Table 1. Using these features, virtual screening of ZINC database was performed, with hits that exhibited an RMSD of less than 0.5 Å were selected for the molecular docking studies. A total of 4809 hits were obtained, based on the developed pharmacophore model.

**Table 1:** The cartesian coordinates of the developed pharmacophore hypothesis.

Pharmacophoric Features	X	Y	Z	Radius
Hydrogen Donor	81.46	40.22	16.95	1
Hydrogen Acceptor	81.95	41.44	18.73	1
Hydrogen Acceptor	80.96	46.17	16.42	1
Aromatic	82.25	43.75	16.31	1
Hydrophobic	87.13	35.74	20.08	1
Hydrophobic	80.46	40.91	14.85	1

### 3.2 Molecular docking studies

The compounds screened by virtual screening were prepared and their docking analysis was conducted against the ROCK2 protein. The binding affinities of all docked compounds were analyzed and then top ten compounds with binding affinities ranging from -9.939 to -9.082 kcal/mol were selected for further analysis (Table 2). The docking scores of the selected compounds suggested that these have potential for inhibiting the function of the ROCK2 protein.

**Table 2:** The binding affinities of the selected compounds along with their structures.

Sr.	Compounds	Glide score (kcal/mol)
1	ZINC287819616	-9.939
2	ZINC513417492	-9.894
3	ZINC112456775	-9.892
4	ZINC376265879	-9.615
5	ZINC373892702	-9.534
6	ZINC40033528	-9.411
7	ZINC12789465	-9.309
8	ZINC219845340	-9.125
9	ZINC21403712	-9.1
10	ZINC43483217	-9.082

### 3.3 Post docking analysis

**Fig 2:**
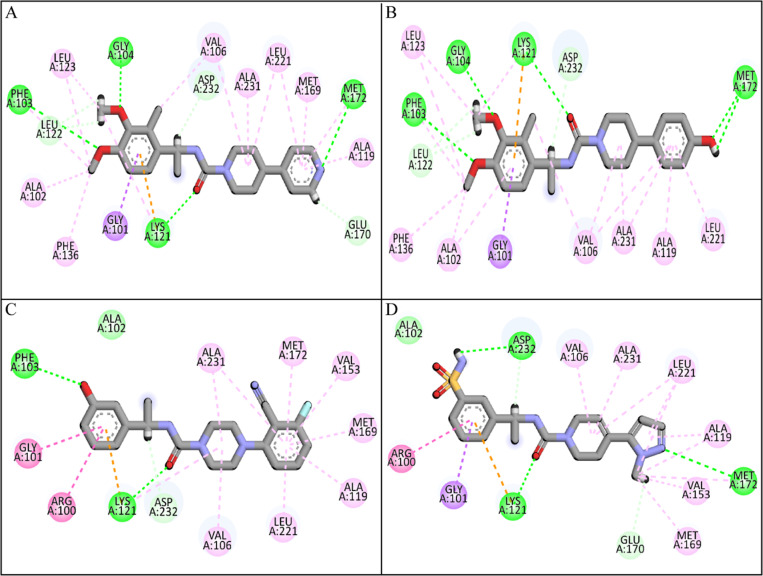
The interactions of the top four compounds with ROCK2 protein. (A) ZINC287819616, (B) ZINC513417492, (C) ZINC112456775, (D) ZINC376265879. Green spheres show conventional hydrogen bonds, gray show carbon hydrogen bonds, purple shows Pi-Sigma interaction, light green shows van der Waal interactions, and magenta shows alkyl interactions.

The docked poses of the selected compounds were analyzed by Discovery Studio client tool to find the molecular interactions. The molecular interactions mainly involved hydrogen bonding, van der Waal interactions, pi-pi stacking, pi-sigma interactions, and alkyl (hydrophobic) interactions. The molecular interactions of each compound helped in determining the binding affinities and their significance. Especially, the hydrogen bonds among ligand and protein atoms play an important role in the strength of protein-ligand complex [45]. **ZINC287819616** formed four conventional hydrogen bonds with Phe103, Gly104, Lys121, Met172, one Pi-Sigma interaction with Gly101, three carbon hydrogen bonds with Asp232, Leu122, Glu170 and eight alkyl interactions with Ala102, Phe136, Leu123, Val106, Ala231, Leu221, Met169, Ala119 (Fig 2A). **ZINC513417492** formed four conventional hydrogen bonds with Phe103, Gly104, Lys121, Met172, two carbon hydrogen bonds with Leu122, Asp232, one Pi-Sigma interaction with Gly101 and seven alkyl interactions with Leu123, Phe136, Ala102, Val106, Ala231, Ala119, Leu221 (Fig 2B). **ZINC112456775** made one van der Waal interaction with Ala102, two conventional hydrogen bonds with Phe103, Lys121, one carbon hydrogen bond with Asp232 and nine alkyl interactions with Gly101, Arg100, Val106, Leu221, Ala119, Met169, Val153, Met172, Ala231 (Fig 2C). Lastly, **ZINC376265879** made one van der Waal interaction with Ala102, one carbon hydrogen bond with Glu170, three conventional hydrogen bonds with Asp232, Lys121, Met172, one Pi-Sigma interaction with Gly101 and seven alkyl interactions with Arg100, Val106, Ala231, Leu221, Ala119, Val153, Met169 (Fig 2D). The molecular interactions of other compounds are shown in Table 3.

**Table 3:** The molecular interactions of the selected compounds with ROCK2 protein.

Sr.	Compound code	Interactions
1	ZINC287819616	**Conventional Hydrogen Bond:** Phe103, Gly104, Lys121, Met172 **Pi-Sigma:** Gly101 **Carbon Hydrogen Bond:** Asp232, Leu122, Glu170 **Alkyl:** Ala102, Phe136, Leu123, Val106, Ala231, Leu221, Met169, Ala119
2	ZINC513417492	**Conventional Hydrogen Bond:** Phe103, Gly104, Lys121, Met172 **Carbon Hydrogen Bond:** Leu122, Asp232 **Pi-Sigma:** Gly101 **Alkyl:** Leu123, Phe136, Ala102, Val106, Ala231, Ala119, Leu221
3	ZINC112456775	**Van der Waal:** Ala102 **Conventional Hydrogen Bond:** Phe103, Lys121 **Carbon Hydrogen Bond:** Asp232 **Alkyl:** Gly101, Arg100, Val106, Leu221, Ala119, Met169, Val153, Met172, Ala231
4	ZINC376265879	**Van der Waal:** Ala102 **Conventional Hydrogen Bond:** Asp232, Lys121, Met172 **Carbon Hydrogen Bond:** Glu170 **Pi-Sigma:** Gly101 **Alkyl:** Arg100, Val106, Ala231, Leu221, Ala119, Val153, Met169
5	ZINC373892702	**Van der Waal:** Gly101 **Conventional Hydrogen Bond:** Met172, Lys121 **Carbon Hydrogen Bond:** Asp232, Glu170 **Alkyl:** Ile98, Phe384, Val106, Arg100, Phe103, Leu123, Phe136, Ala102, Ala231, Leu221, Ala119
6	ZINC40033528	**Van der Waal:** Gly101 **Conventional Hydrogen Bond:** Ala102, Asp232, Lys121 **Pi-Cation:** Met169 **Pi-Sigma:** Leu221 **Alkyl:** Arg100, Val106, Tyr171, Met172, Ala119, Ala231
7	ZINC12789465	**Conventional Hydrogen Bond:** Met172, Lys121, Phe103 **Pi-Sigma:** Gly101 **Alkyl:** Met169, Val106, Leu211, Ala119, Ala231, Phe136, Ala102
8	ZINC219845340	**Conventional Hydrogen Bond:** Lys121, Phe103, Asp232 **Pi-Cation:** Met169 **Pi-Sigma:** Val106 **Alkyl:** Ala119, Met172, Leu221, Ala231, Arg100, Gly101, Ala102
9	ZINC21403712	**Van der Waal:** Gly101 **Conventional Hydrogen Bond:** Ala102, Asp232, Lys121 **Pi-Cation:** Met169 **Alkyl:** Arg106, Ala231, Val106, Ala119, Leu221, Phe384, Ile98, Tyr171
10	ZINC43483217	**Conventional Hydrogen Bond**: Lys121 **Pi-Cation:** Met169 **Pi-Sigma:** Gly101 **Carbon Hydrogen Bond:** Glu170 **Alkyl:** Ala119, Met172, Leu221, Val106, Ala231, Leu123, Ala102, Phe103, Phe136

### 3.4 ADMET analysis

The ADMET and toxicity risks profiles of the selected compounds were analyzed by OSIRIS Property Explorer tool, and it was observed that the predicted values were in the acceptable range (Table 4). The molecular weight plays a vital role in the distribution of a compound within cells, with lower-weight compounds generally able to distribute more easily throughout the body compared to those with higher weights. To address this, a threshold of 500 g/mol was established, and all selected compounds fell within this range. cLogP determines the hydrophilicity of compound, a value of cLogP > 5 indicates poor absorption. The selected hits had cLogP values less than 5, indicating good absorption of compounds. The TPSA relates with the hydrogen bonding of a compound and is a good predictor of bioavailability [46]. TPSA < 160 Å^2^ shows that the compound will have good oral bioavailability [47]. The hits had TPSA values in the range of 63.59 to 118.7 Å^2^. Solubility is also a crucial factor in pharmacokinetics, influencing both the absorption and distribution of a compound. It is typically quantified as the logarithm of the solubility, expressed in mol/dm^3^. This measurement helps to assess how easily a compound dissolves in a solvent, which is vital for its effective utilization in the body and its overall pharmacokinetic profile. The drug score is a comprehensive measure that simplifies several variables, including toxicity risk, molecular weight, logS, and cLogP, into a single, easily comprehensible value. This score is used to evaluate a compound’s overall potential to become a drug. A higher drug score indicates a greater likelihood that the compound could be a viable drug candidate. In essence, the higher the drug score's value, the more likely the compound is to be considered for further drug development [48]. Furthermore, the toxicity profile of compounds was evaluated, and it was observed that the compounds did not show toxicity tendencies except for **ZINC12789465** which showed high risk for mutagenic and reproductive effect. Furthermore, the absorption and distribution profiles of the compounds were predicted by admetSAR web-server (http://lmmd.ecust.edu.cn/admetsar2/). The human intestinal absorption (HIA), Human Oral Bioavailability (HOB), Caco-2 permeability, Blood-brain barrier penetration, and sub-cellular localization of the compounds were predicted, and it was observed that all compounds have the human intestinal absorption. Similarly, all the compounds have the ability to penetrate the blood-brain barrier except for **ZINC219845340**. Lastly, the subcellular localization revealed that all compounds have mitochondrial localization except for **ZINC373892702**, which was localized in lysosome (Table 5).

**Table 4:** The ADMET and Toxicity risks analysis of top ten compounds.

	Pharmacokinetic Properties						Toxicity Risks			
Compounds	MW	cLogP	TPSA	LogS	Drug likeness	Drug score	Mutagenic	Tumorigenic	Irritant	Reproductive effect
ZINC287819616	383	3.44	63.59	-3.44	6.09	0.75	Passed	Passed	Passed	Passed
ZINC513417492	398	4.09	71.03	-3.93	7.17	0.66	Passed	Passed	Passed	Passed
ZINC112456775	368	2.52	79.6	-3.87	1.49	0.70	Passed	Passed	Passed	Passed
ZINC376265879	389	0.35	118.7	-2.04	5.36	0.87	Passed	Passed	Passed	Passed
ZINC373892702	383	3.04	72.92	-3.94	5.13	0.74	Passed	Passed	Passed	Passed
ZINC40033528	374	2.04	104.9	-3.17	-3.09	0.43	Passed	Passed	Passed	Passed
ZINC12789465	382	2.89	59.59	-4.38	-5.29	0.1	High	Passed	Mild	High
ZINC219845340	381	2.03	74.59	-3.35	3.31	0.8	Passed	Passed	Passed	Passed
ZINC21403712	364	2.09	95.67	-3.32	3.31	0.81	Passed	Passed	Passed	Passed
ZINC43483217	382	1.83	66.93	-1.94	0.68	0.72	Passed	Passed	Passed	Passed

**Table 5:** The absorption and distribution profiles of the top ten compounds.

Compounds	HIA	Caco-2	BBB	HOB	Subcellular localization
ZINC287819616	+	+	+	+	Mitochondria
ZINC513417492	+	+	+	–	Mitochondria
ZINC112456775	+	+	+	+	Mitochondria
ZINC376265879	+	–	+	+	Mitochondria
ZINC373892702	+	–	+	–	Lysosome
ZINC40033528	+	–	+	–	Mitochondria
ZINC12789465	+	+	+	–	Mitochondria
ZINC219845340	+	–	–	+	Mitochondria
ZINC21403712	+	+	+	+	Mitochondria
ZINC43483217	+	–	+	–	Mitochondria

### 3.5 Analysis of plausible binding modes

**Fig 3:**
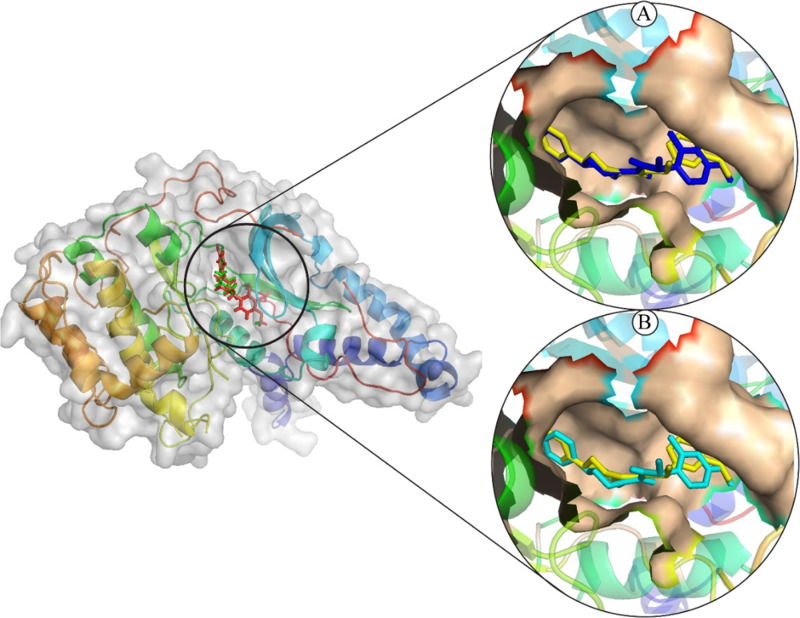
The plausible binding poses of the selected compounds (Cocrystal: yellow sticks). (A) ZINC287819616 (Blue sticks), (B) ZINC513417492 (Cyan sticks).

The plausible binding modes of the selected compounds were observed by alignment on co-crystal ligand. This alignment showed that the docked poses of the hits occupied the same space in binding sites of the ROCK2 as occupied by co-crystal ligand (Fig 3). As a result, the plausible binding modes of the docked hits were evaluated for stability through Molecular Dynamics (MD) simulations.

### 3.6 MD simulation

#### 3.6.1 RMSD.

**Fig 4:**
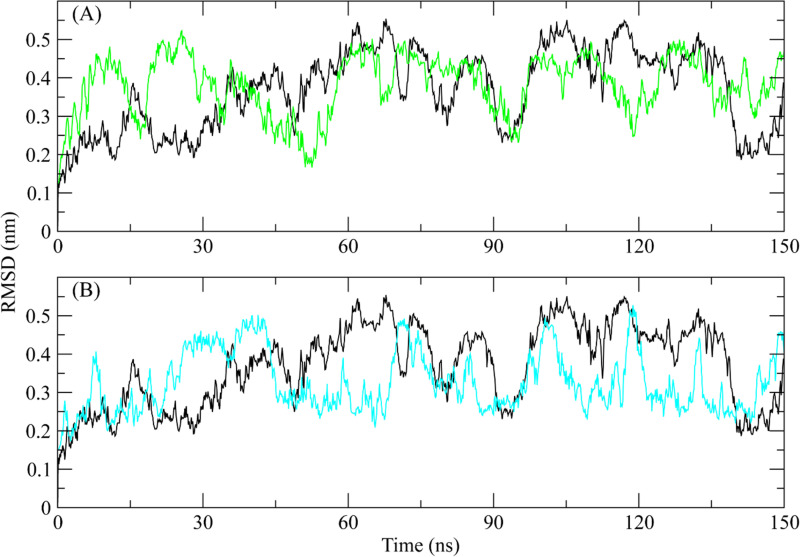
The RMSD of ROCK2 complexes calculated during 150 ns simulation. Black plot shows the RMSD of apo protein (A) ZINC287819616 (Green plot), (B) ZINC513417492 (Cyan plot).

The root mean square deviation of the Cα atoms was measured to evaluate the structural changes of the complexes [49]. The RMSD of the Cα atoms of the apo protein remained between 0.2 and 0.3 nm for the first 30 ns, gradually rising to 0.5 nm at 60 ns. It then exhibited deviations ranging from 0.3 to 0.6 nm until 140 ns, before decreasing to 0.25 nm by the end of the simulation (Fig 4 black plot). On the other hand, the RMSD of **ZINC287819616** complex went gradually to 0.5 nm at 30 ns and then dropped to 0.2 nm at 60 ns, after 60 ns it showed the similar trend as apo protein structure (Fig 4A green plot). The RMSD of **ZINC513417492** displayed variations at several points during the simulation, although it generally stayed within the region of 0.3 nm. It increased to 0.5 nm at 40 ns and subsequently decreased to 0.3 nm at 45 ns (Fig 4B cyan plot).

#### 3.6.2 RMSF.

**Fig 5:**
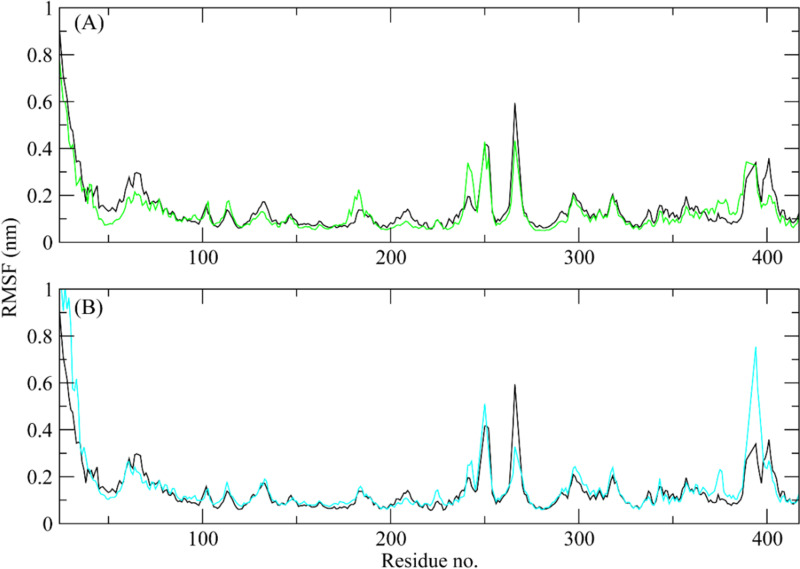
The comparative RMSF plots of the ROCK2 apo protein and in the presence of hit compounds. Black plot shows the RMSF of apo protein (A) ZINC287819616 (Green plot), (B) ZINC513417492 (Cyan plot).

RMSF values were calculated to investigate the protein residues dynamics upon interacting with the ligands [50]. The RMSF plots revealed that most of the residues did not show many fluctuations during simulation as the RMSF values were lower than 0.2 nm, throughout the simulation indicating that the ligands did not exert the fluctuations in the protein. In contrast, the residues in the loop regions showed high fluctuations reaching around 1 nm at N-terminal and up to 0.5 nm at the residues ranging from 240 to 270 (Fig 5). According to the Root Mean Square Fluctuation (RMSF) analysis, the protein-ligand complex exhibited overall stability, as most residues maintained a rigid conformation.

#### 3.5.3. Radius of Gyration (Rg).

**Fig 6:**
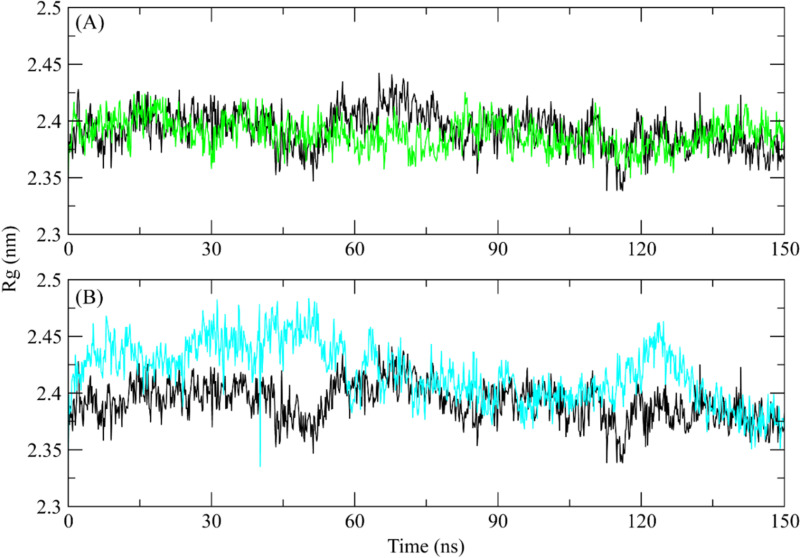
The Rg analysis of the ROCK2 complexes during 150 ns simulation. Black plot shows the Rg of apo protein (A) ZINC287819616 (Green plot), (B) ZINC513417492 (Cyan plot).

To evaluate the structural compactness of the ROCK2 protein when attached to the selected ligand, Radius of Gyration (Rg) study was performed. Higher Rg values demonstrate the unfolding of events during simulation, whereas lower Rg values suggest the compactness of the structure. According to the complex’s Rg plot, the Rg values stabilized between 2.4 and 2.45 nm and stayed in this range over the duration of the simulation. it was observed that the Rg of the **ZINC287819616** showed a similar trend to apo protein (Fig 6A) while the Rg values of **ZINC513417492** complex were slightly higher than apo protein till 60 ns and then showed similar values to apo structure (Fig 6B). The stable Rg values indicate that the protein structure remained compressed when it was coupled to the ligand throughout simulation.

#### 3.5.4. SASA.

**Fig 7:**
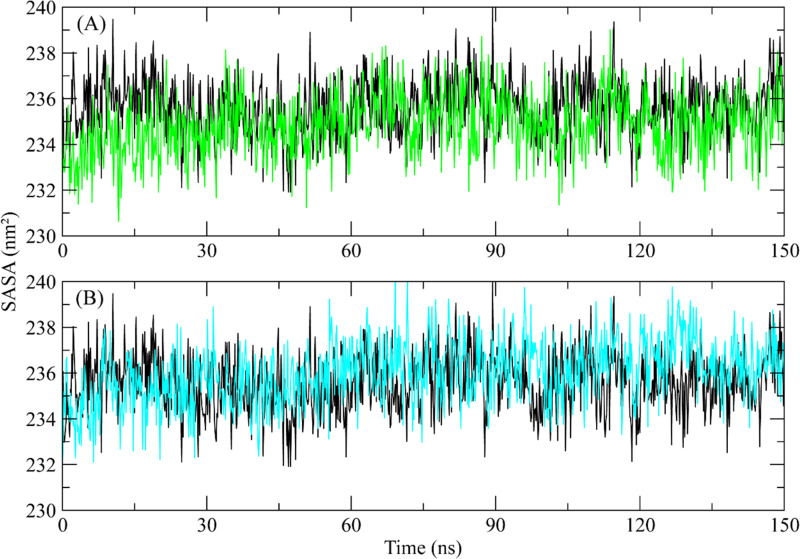
The SASA plot of the ROCK2 complexes with selected compounds. Black plot shows the SASA plot of apo protein (A) ZINC287819616 (Green plot), (B) ZINC513417492 (Cyan plot).

Moreover, the SASA (Solvent Accessible Surface Area) analysis was conducted. The purpose of the SASA was to determine the solvent accessible area of the protein during simulation and to look for any conformational changes. According to the study, the protein’s original SASA value was around 234 nm^2^, and it stayed inside this range throughout the whole simulation in both complexes and the apo protein structure (Fig 7). The SASA values indicated that the protein structure did not face confirmational changes during the simulation.

#### 3.5.5. Hydrogen bonding.

**Fig 8:**
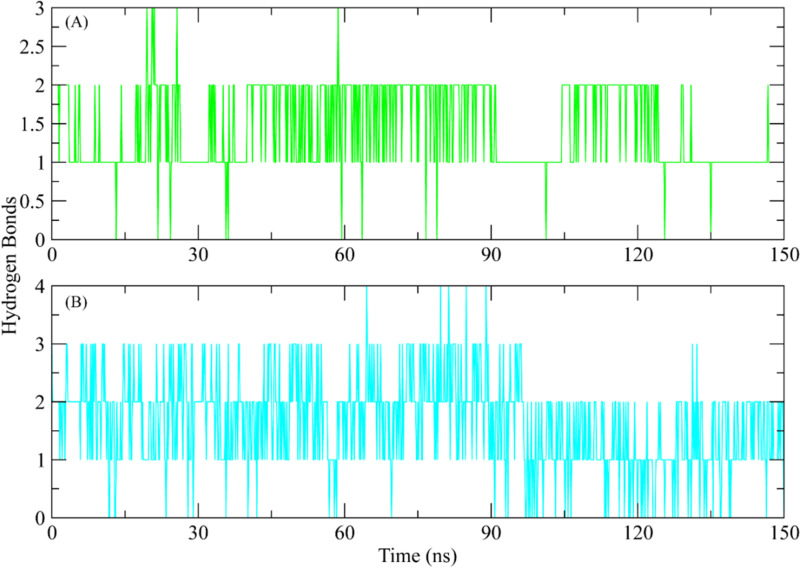
The observed hydrogen bonds during simulation in ROCK2 complexes. (A) ZINC287819616 (Green plot), (B) ZINC513417492 (Cyan plot).

Hydrogen bonding is essential for the protein-ligand combination to remain stable. Consequently, the ligand hydrogen bonds, and active site residues were calculated. The hydrogen bonding plots indicate that **ZINC287819616** made at least 2 hydrogen bonds with protein. The number of hydrogen bonds exceeded 3 at some frames (Fig 8A). On the other hand, ZINC513417492 made up to 3 hydrogen bonds till 90 ns and then the number of hydrogen bonds reduced to 2 in later part of simulation (Fig 8B).

#### 3.5.7 PCA.

**Fig 9:**
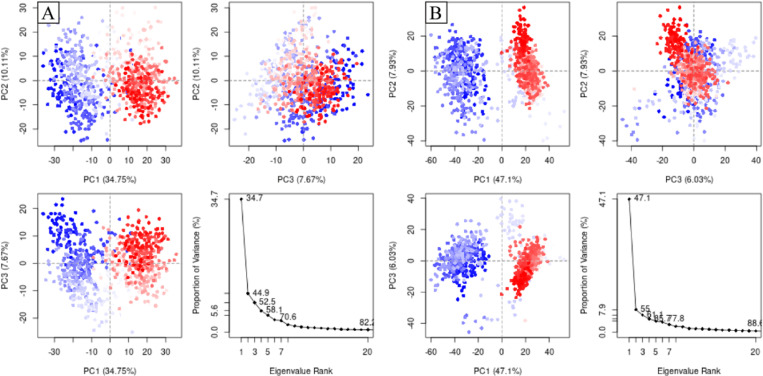
The principal component analysis to calculate the variance in protein clusters upon binding of the compounds. (A) ZINC287819616, (B) ZINC513417492.

The principal component analysis (PCA) was performed to calculate the variance percentage in protein clusters upon binding of the compounds. In the analysis, the proportion of variance was plotted against the eigen value rank to calculate the movements in different hyperspaces. The dominant movement was observed in the first five eigenvectors in both complexes. The first five eigenvectors in ZINC287819616 complex showed the eigenvalues of 34.7, 10.11, 7.67, 5.6, and 6.2% respectively. The total variation was 82.2%. The highest variation was observed in PC1 which recorded 34,75% fluctuations during the simulation (Fig 9A). Similarly, in ZINC513417492 complex, the highest fluctuations were observed in PC1 with a value of 47.1% and the overall variation was 88.6% which indicated that this compound exerted variations in protein clusters during the simulation (Fig 9B).

## 4. Discussion

The discovery of novel therapeutic agents is a complex and resource-intensive process that frequently necessitates the integration of advanced computational methodologies with experimental techniques. Computational screening approaches have gained prominence in recent years as efficient tools for identifying potential drug candidates [51,52]. Among various drug targets, Rho-associated protein kinase 2 (ROCK2) has emerged as a promising target for a range of diseases, including cancer, cardiovascular disease, and neurological disorders [4,53]. This study emphasizes the computational screening of the ZINC database to identify potential ROCK2 inhibitors by employing computational drug design techniques.

Pharmacophores were used to virtually screen the ZINC database to identify the best binding modes. The ROCK2's active site was then docked to the screened hits of compounds. Pharmacophore modeling identifies key chemical features needed for an inhibitor to interact with the target, resulting in a structural blueprint for virtual screening [54]. A ligand-based pharmacophore model was developed by using a ROCK2 protein’s chemical characteristics. co-crystal ligand (5YS) (PDB ID: 7P6N). The virtual screening model was developed using the four pharmacophoric properties of co-crystal ligand. A ligand-based virtual screening of the ZINC database was performed based on these characteristics, and the 4809 hits that met the screening criteria were chosen and prepared.

The molecular docking studies of the screened hit compounds were conducted to the ROCK2 protein by standard precision mode of glide tool to predict their binding affinities. Molecular docking is a useful approach in drug development because it allows scientists to predict how prospective drug candidates will interact with proteins. This data can be used to aid the development and optimization of novel drugs molecules. This analysis indicates the importance of molecular interactions of the compounds with the protein which can help to design the new drug candidates and to optimize the existing drugs [55–57]. The top ten compounds with binding affinities ranging from -9.939 to -9.082 kcal/mol were selected for further study.

The assessment of ADMET characteristics and toxicity risks for the selected compounds revealed predicted values within an acceptable range. Evaluating the ADMET properties is a crucial aspect of the drug development process. It enables the prediction of potential toxicity, behavior, and outcomes of a proposed drug within the human body. Understanding these properties aids in making informed decisions about the safety and efficacy of drug candidates during the development phase [58–60].

The plausible binding modes of the two hits were examined with alignment of the co-crystal ligand. Consequently, the aligned modes were then subjected to MD simulations for the protein dynamics and structure stability analysis. MD simulations serve as an effective tool for understanding the stability of protein-ligand complexes [61]. The MD simulations analysis suggests that the selected compounds remained stably bound to the target protein.

This comprehensive approach, which integrates detailed interaction analysis with dynamic simulation data, sets a new benchmark in the field by reducing experimental trial and error and accelerating lead identification. Ultimately, our methodology provides a promising solution for the development of targeted ROCK2 inhibitors, offering potential therapeutic avenues for conditions such as cancer metastasis, cardiovascular disorders, and neurodegenerative diseases by effectively mitigating the deleterious effects of ROCK2 dysregulation. On contrast Computational methods have some drawbacks such as the variability in results produced by different tools for the same analysis, making it necessary to approach findings with caution and validate them through additional investigation in wet labs. Considering our findings about the bioactivity of certain compounds, more study into structure-based lead optimization is required.

## 5. Conclusion

In current study, we have identified hits from different databases that have potential to inhibit the activity of ROCK2 protein. The binding affinities of the screened hits were determined by molecular docking which indicated the strong interactions between ligand and protein. Further, the protein dynamics and confirmational changes were analyzed by MD simulation which showed no major changes in the protein and stability of bound ligands with proteins. The findings of the study concluded that the identified hits can be potential leads to counter the activity of ROCK2 protein.
