# Expression of Concern: Discovery of ROCK2 inhibitors through computational screening of ZINC database: Integrating pharmacophore modeling, molecular docking, and MD simulations

**DOI:** 10.1371/journal.pone.0362152

**Published:** 2026-11-05

**Authors:** The PLOS One Editors

The *PLOS One* Editors issue this Expression of Concern because of concerns about the integrity and/or reliability of the peer review process.

Readers are advised to interpret the article [1] with consideration of this issue. In following up on this matter, PLOS did not evaluate the article’s integrity or scientific validity.

The Editors do not have concerns regarding the authors’ conduct during the peer review process.
